# Comparison Between Real-Time Ultrasound-Guided Percutaneous Tracheostomy and Surgical Tracheostomy in Critically Ill Patients

**DOI:** 10.1155/2022/1388225

**Published:** 2022-09-25

**Authors:** Hyun Tag Kang, Shin Young Kim, Min Ki Lee, Seung Won Lee, Aerin Baek, Ki Nam Park

**Affiliations:** ^1^Department of Otorhinolaryngology-Head and Neck Surgery, Soonchunhyang University, Bucheon, Republic of Korea; ^2^Department of Internal Medicine, Soonchunhyang University, Bucheon, Republic of Korea

## Abstract

**Background:**

Ultrasound-guided percutaneous dilatational tracheostomy (US-PDT) has been adapted for use in intensive care units (ICU). US-PDT is comparable to bronchoscopy-assisted tracheostomy. However, compared to surgical tracheostomy (ST), its safety and effectiveness have not been well studied.

**Objectives:**

To determine the efficacy and safety of US-PDT compared to ST.

**Materials and Methods:**

A total of 90 patients who underwent US-PDT (*n* = 36) or ST (*n* = 54) between July 2019 and September 2020 were enrolled. US-PDT was performed in the ICU without a surgical assistant or bronchoscope. Data were collected retrospectively and analyzed regarding clinical characteristics, procedure times and details, complications, and mortality rate.

**Results:**

The success rate of US-PDT was 97.4% and the procedure time was shorter than ST (5.2 ± 3.1 vs. 10.5 ± 5.0 min). There were no significant differences in clinical characteristics and procedure details. There was no procedure-related mortality in either of the groups.

**Conclusions:**

US-PDT is time-efficient and as safe as ST. Based on our results, US-PDT may be considered a potential alternative to ST in high-risk patients and in those who cannot be transported.

## 1. Introduction

Tracheostomy is one of the most commonly performed procedures in intensive care units (ICUs) and may become more popular as the demand for intensive care services increases [1]. Open surgical tracheostomy (ST) is the standard procedure but has a relatively high incidence of peristomal infections and perioperative bleeding [2]. Percutaneous dilatational tracheostomy (PDT) was introduced in 1985 [3] and has since become a common bedside procedure. Compared to open STs, PDTs have the advantages of lower risk of wound infection, less bleeding-related mortality, shorter procedure time, and cost-effectiveness [4, 5].

Bronchoscopy is commonly used during PDT to guide needle insertion into the trachea, verify the cannula's location, and enhance safety [6]. However, bronchoscopy-guided PDT has several limitations relating to the precise identification of cervical anatomical structures and the prevention of complications, such as vessel injury [7].

Ultrasound assistance has several potential advantages, including portability, safety, identification of cervical vasculature, and localization of tracheal puncture [7–9]. Several studies have demonstrated that the use of real-time ultrasound may improve the rate of first-pass puncture and puncture accuracy and reduce procedure time and complications [6, 9]. However, the available data are limited to preliminary studies comparing open ST and ultrasound-guided percutaneous dilatational tracheostomy (US-PDT) [10]. Hence, this retrospective study was designed to compare the safety and efficacy of US-PDT to ST at the ICU bedside in critically ill patients.

## 2. Materials and Methods

### 2.1. Patients

A single-center retrospective study was conducted on 90 patients who underwent open STs (*n* = 54) or US-PDTs (*n* = 36) in the ICU between July 12, 2019, and Sep 30, 2020. The study protocol was approved by Institutional Review Board and the requirement for informed consent from the patients was waived. ST and US-PDT were conducted by a single surgeon with over 15 years of tracheostomy experience.

The exclusion criteria were age ˂18 years; tracheostomy site infection; previous history of irradiation or tracheostomy; severe coagulopathies (platelet count <20,000/mL) or hemodynamic instability; and emergent tracheostomy. All procedures were performed electively and, an informed consent for ST and US-PDT (including the possibility of ST conversion) was obtained before the procedures.

### 2.2. Ultrasound-Guided Percutaneous Dilatational Tracheostomy

**Figure 1:**
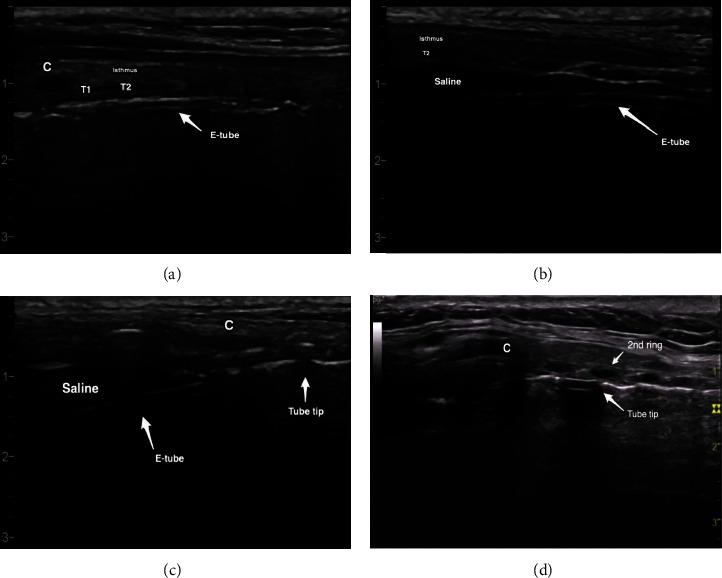
Endotracheal tube repositioning for percutaneous dilatational tracheostomy under ultrasound guidance. (a) Double-linear hyperechogenecity revealed the ET tube. (b) Anechoic filling and the ET tube were identified beneath the tracheal cartilage after saline ballooning. (c) Saline ballooning superior to the cricoid cartilage and pushing of the tracheal cartilage anteriorly by the ET tube tip were identified after pull back. (d) The ET tube tip was identified just superior to the 2nd tracheal ring in another patient. C: cricoid cartilage, ET tube: endotracheal tube, *T1 and T2*: 1^st^ and 2^nd^ tracheal ring, respectively.

**Figure 2:**
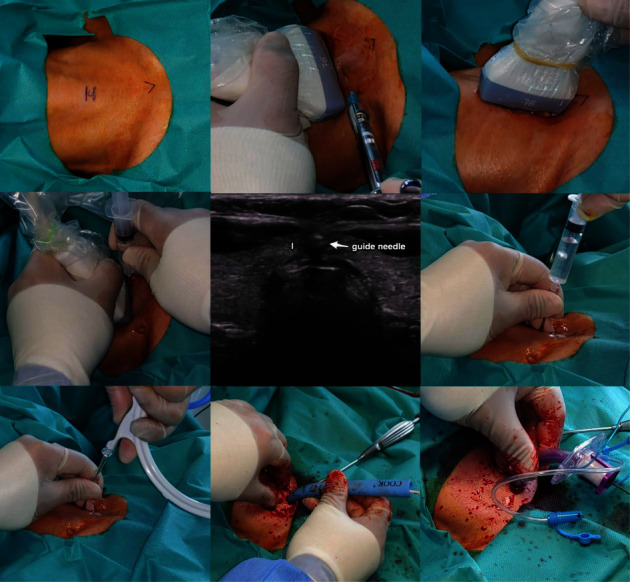
Real time ultrasound-guided percutaneous dilatational tracheostomy. After marking the cricoid cartilage and sternal notch, local anesthesia was injected superficial to the thyroid isthmus. Under sagittal conversion of the ultrasound probe, the ET tube was repositioned to an appropriate level to prevent tube puncture. The guide needle was inserted carefully to avoid damaging the cervical vasculature in the axial ultrasound view, followed by air regurgitation and insertion of the guidewire. The T-tube could be inserted along the guidewire following stepwise multiple dilatations using the Ciaglia Blue Rhino tracheostomy kit. I: thyroid isthmus, ET tube: endotracheal tube, T-tube: tracheostomy tube.

The procedure was performed without a surgical assistant, and only one medical staff was required to readjust the endotracheal tube (ET tube). Real-time ultrasound was used to determine the appropriate tracheal cartilage level. Under ultrasound guidance, ET tube repositioning was performed to insert a guide needle and prevent tube fenestration. After deflation of the tube balloon, the ET tube tip was identified and repositioned using a combination of three techniques: saline ballooning [11], double-linear tube hyperechogenicity just under the tracheal cartilage, and pushing of the tube tip into the trachea anteriorly (Figure 1). After the local injection of 2% lidocaine mixed with 1 : 100,000 epinephrine, the guide needle was inserted under ultrasound guidance through the thyroid isthmus, and a guide wire was inserted along the needle after identification of air regurgitation. A skin incision less than 1.5 cm in size was made, and stepwise multiple dilatations were performed using the Ciaglia Blue Rhino tracheostomy kit (Cook Medical Inc., Bloomington, IN, USA). After the removal of the dilatational tube, a tracheostomy tube (T-tube) appropriate for the sex of the patient (internal diameter = 7 and 8 mm for females and males, respectively) was inserted using the guidewire. The details of the procedure are shown in Figure 2.

### 2.3. Surgical Tracheostomy

ST was performed using a conventional method as follows: horizontal skin incision, vertical dissection of the linea alba cervicalis, exposure and division of the thyroid isthmus, and rectangular window formation between the 2^nd^ and 3^rd^ tracheal rings. The thyroid isthmus was divided to identify an appropriate tracheal level under direct vision and hemostasis was achieved.

### 2.4. Data Collection

The medical records of all included patients were reviewed retrospectively. We analyzed the demographic characteristics and clinical parameters, including ET tube and T-tube inner diameters, body mass index (BMI), intubation period, repositioned depth of the ET tube at the corner of the mouth, indications for and anatomical difficulty of tracheostomy, tracheal deviation, distance from the cricoid cartilage to the sternal notch, and limitations of neck extension. Before the tracheostomy, we measured the tracheal width and distance between the skin and the second tracheal ring using ultrasound. The procedure time was defined as that from skin incision to T-tube insertion in ST, and as that from ET tube repositioning under real-time ultrasound guidance to T-tube insertion in US-PDT. After all procedures, the tracheal lumen and carina were identified using a portable fiberoptic scope, to confirm successful tube insertion and detect tracheal wall abrasions. Postprocedure chest X-rays were used to check for subcutaneous emphysema, pneumothorax, and pneumomediastinum. Immediate and delayed postoperative complications were investigated at least 3 months after tracheostomy. The length of ICU stay and causes of hospital mortality were also investigated.

### 2.5. Statistical Analysis

SPSS Statistics software (version 26.0; IBM Corp., Armonk, NY, USA) was used for the analyses. Student's *t* test and the chi-square test were used to analyze numeric and nonnumeric parameters, respectively.

## 3. Results

The demographics and preprocedural data are presented in Table 1. There were no differences in age, sex, or BMI between the two groups. The intubation periods were 14.4 ± 9.6 and 11.5 ± 7.2 days in the ST and US-PDT groups, respectively. Anatomical difficulties were present in four patients in each group, while limited neck extension was present in one and three patients in the ST and US-PDT groups, respectively. ET tube sizes were not different between the two groups. ST was difficult in one obese patient because of a very short neck (2 cm from the lower cricoid border to the sternal notch), while US-PDT was difficult in two patients because the trachea was too stiff for dilatation. There were no significant differences in anatomical difficulty, limitation of neck extension, or procedure difficulty between the groups. The success rate of US-PDT in this study was 97.4% (37/38). One patient in the US-PDT group required ST conversion, and a T-tube was successfully inserted within 1 minute.

Procedure-related data and complications are shown in Table 2. The distance between the lower border of the cricoid cartilage and the sternal notch was measured after the Rose position and was not different between the groups. Tracheal depth and width on ultrasound also showed no differences between the groups. Procedural time in the US-PDT group (5.2 ± 3.1 minutes) was significantly shorter than in the ST group (10.5 ± 5.0 minutes), despite ultrasound-guided tube repositioning (*p* < 0.001). The repositioned ET tube depth in the US-PDT group was 18.2 ± 0.8 and 17.5 ± 1.3 cm in males and females, respectively (*p* < 0.078). The estimated blood loss and inserted T-tube sizes were not different between the groups. Desaturation episodes with SpO_2_ <90% occurred in three patients (two and one in the US-PDT and ST groups, respectively) during the procedures, but there were no life-threatening events.

During US-PDT, the guide wire kinked during the last dilatation in two patients. Reinsertion of the guide wire was feasible because early dilated lumen could be identified easily. The tracheal tube was inserted into the false lumen in one patient in both groups, and immediate removal followed by reinsertion was successfully performed without any life-threatening desaturation episodes. Posterior tracheal wall abrasion was observed in one patient in both groups; however, there were no cases of posterior wall rupture or tracheoesophageal fistula. Bleeding from the tracheostomy was observed in 11 patients (9 and 2 in the ST and US-PDT groups, respectively) and was controlled by conservative hemostasis, including bedside gauze packing and electrocauterization. Post-T-tube insertion occlusion occurred in one patient, while infection occurred in two patients in the ST group.

There were no cases of deteriorating subcutaneous emphysema, pneumomediastinum, or pneumothorax in either group. The mean ICU stay was longer in the ST group (30.2 days) than US-PDT group (16.4 days) (*p*=0.014). The hospital mortality rate was 21.1% and 10.5% in the ST and US-PDT groups, respectively. There was no tracheostomy-related mortality and deaths only occurred due to the progression of underlying diseases.

## 4. Discussion

Real-time US-PDT was introduced in 1999 [12] and has since then emerged as a useful tool. Several studies have reported its effectiveness and safety [9, 13, 14]. Our results demonstrated that US-PDT was an effective method, with a high (97.4%) success rate similar to a recent randomized controlled noninferiority trial [7]. Only one case required surgical dilatation with mosquito forceps after the withdrawal of the guidewire. US-PDT has a relatively long learning curve [15] because the identification of an endotracheal cuff balloon is difficult and easy to puncture [9]. We suggested some technical tips to identify and readjust the endotracheal tube repositioning in this study, however, physicians should be trained in US and neck anatomy before the beginning of US-PDT, aware of the possibility of tube puncture, and be prepared for procedure failure and immediate conversion to open tracheostomy.

In terms of procedural time, US-PDT was performed faster (5.3 minutes) than ST in this study. This was in agreement with a recent meta-analysis that reported mean procedure times of 8.6–41.5 and 7.3–24.9 min for ST and US-PDT, respectively [16]. However, our US-PDT procedure time seemed to be slightly shorter than in previous reports because the preparation time and ultrasound guidance were not considered.

There were no tracheostomy-related mortalities or major complications that required surgical intervention in this study. The majority of the perioperative complications were minor and could be managed with supportive care. The complication rates were not significantly different between the two groups and were in agreement with previously reported rates of 6–66% [17]. Although the duration of ICU stay was based on the physician's preference rather than the procedure, the group difference therein suggested that the patients in the ST group had a more severe disease course than the US-PDT group patients. PDT has been reported to have an advantage in terms of postprocedure bleeding [18, 19]; however, stomal bleeding was not significantly different between the groups in this study, probably because of meticulous hemostasis during ST and the exclusion of patients with a bleeding tendency.

US-PDT has many potential advantages over ST; it can be performed under the indirect vision and is more comfortable because the bed is wider than the operating table. Tracheostomy itself is associated with the significant aerosol generation and puts medical staff at risk for bacterial and viral transmission [20]. Indirect vision may help prevent the aerosol contamination that can occur even through a face shield. Moreover, US-PDT requires less essential medical staff and may be associated with lower rates of infectivity during the current pandemic era.

Boran et al. reported that PDT with a flexible light wand was safer, quicker, and more effective compared to ST [18]. Our study demonstrated that real-time US-PDT obviates the need for adjunctive procedures and the risks associated with transportation to the operating room and is safe and can be performed more quickly than ST. Although PDT was reported to be cheaper than ST, by about 450 USD [19], US-PDT is in fact more expensive, by 220 USD, in the Korean health insurance system. Patient copayment is also more expensive, by about 10 USD because of the price of the tracheostomy kit.

Compared to bronchoscopic PDT, US-PDT has several following advantages: accurate tracheal incision level; greater availability of ultrasonography than bronchoscopy equipment in the ICU; shorter procedure length; less labor intensive to clean equipment; the need for fewer medical staff than bronchoscopy [7, 9]. In addition, a lower complication rate has been reported in US-PDT compared to bronchoscopy, especially regarding hemorrhage because of US examination to identify the vascular structures in the anterior tracheal area [9]. In our institution, bronchoscopy PDT had been tried for critically ill patients, however, US-PDT has been introduced and has become a preferred method after several low-level tracheostomy and major hemorrhages.

This study had several limitations, the most notable of which was the retrospective design and the small number of cases. In addition, long-term complications, such as tracheal stenosis, were not evaluated in this study because most of the patients were referred to other hospitals after tracheostomy and follow-up were difficult. Despite these limitations, to our knowledge, this was the first study to evaluate the efficacy and safety of real-time US-PDT performed by a head and neck surgeon in an ICU. US-PDT had similar safety and effectiveness but with a shorter procedural time and maybe a potential alternative to ST, especially in high-risk patients and those who cannot be transported. Larger prospective studies or randomized control studies are needed to further evaluate the safety and effectiveness of US-PDT.

## Figures and Tables

**Table 1:** Demographics of the patients undergoing surgical tracheostomy (ST; *n* = 54) and ultrasound-guided percutaneous dilatational tracheostomy (US-PDT; *n* = 36).

	ST	US-PDT	*p* value
Age (y)	64.2 ± 15.8	60.8 ± 12.1	0.154
Sex (male: female)	20 : 34	11 : 25	0.652
Body mass index (kg/m^2^)	21.4 ± 5.1	22.5 ± 5.0	0.317
Intubation period (days)	14.4 ± 9.6	11.5 ± 7.2	0.129
Anatomical difficulty	4	4	0.709
Limited neck extension	1	3	0.298
Difficult procedure	1	2	0.561
Endotracheal tube size (mm)	7.6 ± 0.5	7.8 ± 0.3	0.104

**Table 2:** Procedure details and related complications by group.

	ST	US-PDT	*p* value
Cricoid to sternal notch (cm)	4.5 ± 1.1	4.9 ± 1.2	0.118
Tracheal depth (mm)	7.2 ± 3.0	7.4 ± 3.0	0.684
Tracheal width (mm)	24.1 ± 3.2	23.4 ± 4.0	0.379
Procedure time (min)	10.5 ± 5.0	5.2 ± 3.1	**<0.001**
USG duration (min)		4.0 ± 1.6	
Repositioned ET tube depth (cm)		Male 18.2 ± 0.8 Female 17.5 ± 1.3	0.078
Estimated blood loss (mL)	4.0 ± 2.9	4.9 ± 5.2	0.296
Tracheostomy tube size (mm)	7.6 ± 0.5	7.7 ± 0.5	0.450
Complications	11 (20.3%)	4 (11.1%)	0.387
False lumen insertion	1	1	0.811
Bleeding	9	2	0.084
Accidental decannulation	1	0	0.412
Tube obstruction	1	0	0.412
Infection	2	0	0.243
Length of ICU stay (days)	30.2 ± 26.8	16.4 ± 14.5	**0.014**
Hospital mortality, *n* (%)	11 (21.1%)	4 (10.5%)	0.181

ST: surgical tracheostomy; US-PDT: ultrasound-guided percutaneous dilatational tracheostomy; USG: ultrasonography; ET tube: endotracheal tube; ICU: intensive care unit.

## Data Availability

The retrospective data used to support the findings of this study are restricted by the Institutional Board Review of Soonchunhyang University Bucheon hospital in order to protect the patient privacy. Data are available from corresponding author (KN Park, man7140@gmail.com) for researchers who meet the criteria for access to confidential data.
